# Exploring the Use of a Learning-Based Exergame to Enhance Physical Literacy, Soft Skills, and Academic Learning in School-Age Children: Pilot Interventional Study

**DOI:** 10.2196/53072

**Published:** 2024-02-23

**Authors:** Aurelie Goncalves, Florence Lespiau, Gaëtan Briet, Eugénie Vaillant-Coindard, Angèle Palermo, Elsa Decobert, Nathan Allegret-Bourdon, Elodie Charbonnier

**Affiliations:** 1 APSY-v University of Nîmes Nîmes France

**Keywords:** learning support, exergaming, physics playground, educational games, primary school, children

## Abstract

**Background:**

There is ample evidence that most children do not perform enough physical activity (PA). To address this major public health problem, the French government implemented 30 minutes of daily PA (DPA) at schools but did not provide any supplemental resources or concrete guidance. Considering both children’s interest in video games and the need for teachers to complete their curriculum, the use of a learning-based exergame that combines PA and learning appears particularly relevant.

**Objective:**

The first objective of this study was to evaluate the feasibility of implementing 30 minutes of DPA through exergaming among school-age children. The second objective was to examine the effects of an exergaming program on physical literacy, academic learning, and soft skills (motivation, self-efficacy, and concentration).

**Methods:**

This interventional study had a pre-post design and used the Play LÜ exergame platform. The study included 79 children with a mean age of 8.9 (SD 1.2) years from grade 2 (7 years old) to grade 5 (11 years old). Play LÜ requires players to throw balls against a wall to reach a target or to activate an object and provides an interactive game area for educational activities linked to specific learning themes. After a 4-session familiarization phase during which the teachers chose to prioritize mathematics learning in 30-minute DPA sessions, students took part in DPA sessions over a period of 3 weeks with Play LÜ and a motor skills circuit behind the LÜ setup to keep them continuously active. All sessions were carried out by PA specialists. Each session started with a warm-up using the Grööve application, continued with main activities promoting mathematics learning adapted to each grade level, and ended with a 3-minute meditation for returning to a calm and serene state using the Gaïa application. Before (T0) and after (T1) the program, students completed a self-evaluation booklet to assess their levels of physical literacy, academic performance, and soft skills.

**Results:**

The implementation of this exergaming program was welcomed by the school’s administration, teaching staff, and parents. After the program, we observed increased scores for physical literacy (difference +2.6, percentage change +3.6%; *W*=933.0; *P*=.002; *r*_rb_=−0.39, 95% CI −0.58 to −0.16) and motivation in mathematics (+0.7, +9.8%; *W*=381.5; *P*=.005; *r*_rb_=−0.44, 95% CI −0.66 to −0.16). In addition, it is important to note that some measures progressed differently across learning levels and age groups.

**Conclusions:**

The study results indicate positive impacts of learning-based exergaming on physical literacy and motivation in mathematics among school-age children.

## Introduction

### Background

Regular exercise and physical activity (PA) have been shown to benefit children’s physical and social health, as well as their academic performance [1-3]. For children, the World Health Organization (WHO) recommends a minimum PA practice of 60 minutes of daily moderate-to-vigorous PA [4]. Yet, in France, only 41.8% of children reach these recommendations [5]. In other words, nearly 6 out of 10 children are physically inactive. In this context, the French national health strategy [6] has set as a major objective the implementation of a comprehensive policy of prevention and health promotion. As a result, since September 2022, primary schools have been required to provide 30 minutes of daily PA (DPA) to promote PA and encourage the development of children’s motor skills and physical abilities. Distinct from the teaching of physical education (PE), the 30 minutes of DPA can take a variety of forms, adapted to the context of each school. They can be split up and combined over the various school and extra-curricular periods. This was extended to all elementary schools after 2 years of testing in 11,000 volunteer schools [7].

Given the significant amount of time children spend in school throughout their childhood, schools represent an ideal setting to achieve maximum impact with regard to improving PA levels [8,9]. Furthermore, several studies have suggested that combining PA with academic activities can improve children’s health and cognitive functioning, which could subsequently lead to an improvement in children’s academic performance [10-12]. Attention, in particular, which is a prerequisite for learning, is often targeted during classroom-based PA [13]. However, other variables, such as motivation and self-efficacy (referring to the child’s perception of his or her capabilities), are well known to influence children’s school performance and acquisition [14-17] and could be influenced by a more entertaining approach to learning via exergaming.

Despite all the benefits of implementing regular PA at school, in a systematic review, Nathan et al [18] highlighted several barriers, such as environmental context and resources with “a lack of time in the curriculum;” goals with “competing curriculum demands of other subjects” or “physical activity considered a lower priority than other subjects;” and beliefs about capabilities, such as a lack of teacher expertise and confidence in delivering PA, and intentions with “a lack of teacher motivation to implement PA” [18]. By contrast, the authors also mentioned several facilitators. Among them, the knowledge domain was indicated to play a facilitating role, for example, “sufficient knowledge about PA and health to effectively conduct PA” [18]. This dimension could be explored through the notion of physical literacy (PL), which corresponds to “the motivation, confidence, physical competence, knowledge, and understanding to value and take responsibility for engagement in physical activities for life” [19]. Indeed, PL is particularly important in early childhood, a crucial period for the development of fundamental movement skills [20] and the adoption of PA habits. Physically literate individuals are more physically active, spend more time playing sports, and are less sedentary. PL is a multi-level concept that is increasingly taken into consideration in the field of public health as it is a key determinant of PA habits across the lifespan [21].

One of the factors behind children’s low levels of PA and high levels of sedentary behaviors is screen time use. Indeed, 71.7% of French boys and 58.5% of French girls aged 6 to 10 years have more than 2 hours a day of screen time [5]. Children aged between 8 and 12 years have 1.5 hours of daily screen time attributable to video games [22]. Over the last decades, video games have emerged as one of the most popular forms of global entertainment. Given children’s keen interest in video games, it seems particularly appropriate to use gamification to encourage PA. For staying active while enjoying the pleasures of video games, a worthwhile alternative is exergaming. Indeed, exergaming or active video gaming requires bodily movements to play the game and encourages PA, with a focus on children’s interest in the game’s dynamics and stimulation. Our approach to exergaming takes into account a health dimension and can be associated with the conceptualization proposed by Oh and Yang [23], defining an exergame as “a video game that promotes (either via using or requiring) players’ physical movements (exertion) that is generally more than sedentary and includes strength, balance, and flexibility activities.”

Given that children spend most of their time at school, that they have a particular appeal for video games, and that exergaming seems to have beneficial effects on school learning [24], the use of exergaming at school appears to be an ideal solution for promoting PA, PL [25-27], and learning [28]. Furthermore, it appears that the use of a technology-based learning environment at school can increase soft skills, such as motivation and concentration on academic tasks [29]. Similarly, it has been shown that incorporating technology into an instructional intervention can improve students’ sense of self-efficacy [30], which is a key variable for academic learning. Exergames, in particular, have been found to promote cognitive functions, motor skill training, enjoyment, and motivation to play among school-age children [31], and improve self-efficacy over traditional exercises [32]. Supporting this idea, it has been shown that exergaming (eg, Nintendo Wii Games [33]) incorporated into PE classes combined with health messages has a higher potential to enhance PA-related attitudes and behaviors than regular PE classes, especially in elementary school children [27]. An interesting exergaming tool for reconciling learning and DPA is the Play LÜ exergame platform (LÜ Interactive Playground) [34]. This technology can be used to change the traditional sports-school atmosphere into an interactive learning environment through interactive wall projection and a synchronized sound system. LÜ Playground activities are designed to improve the learning of children and adolescents by allowing them to respond to questions in specific fields (eg, mathematics, history, and natural sciences) by throwing balls against an interactive wall. This tool would therefore allow the practice of PA within non-PE curricula and thus ensure the 30 minutes of DPA among primary school children. Moreover, given the associations among cognitive functioning, soft skills, learning, and PA demonstrated in the literature, it appears essential to assess whether an exergaming program can improve these different variables.

### Objectives, Research Questions, and Hypotheses

The first objective was to study the feasibility of implementing 30 minutes of DPA through exergaming. Given that exergaming combines the interests of children (for video games) and teachers (for learning and respecting the curriculum) while promoting PA, we hypothesized that it will enable effective implementation of the 30 minutes of DPA in schools.

The second objective of this study was to evaluate the effects of an exergaming program on PL, academic learning, and soft skills (motivation, self-efficacy, and concentration). We hypothesized that implementing a DPA program involving exergaming on a specific academic course combined with information on health-promoting behaviors daily could increase children’s PL (hypothesis 1) and increase academic performance (hypothesis 2). Indirectly, allowing students to work on an academic subject more entertainingly through exergaming could improve students’ motivation in the academic discipline (hypothesis 3), their sense of self-efficacy in the subject (hypothesis 4), and their concentration in class and the academic subject (hypothesis 5).

## Methods

### Population

This study was conducted with children aged 7 to 11 years as part of the implementation of the 30 minutes of DPA policy. The study was designed as an interventional study with a pre-post design. It included children from grade 2 (7 years old) to grade 5 (11 years old) in a mid-sized city school in the southern part of France, who had never benefited from any intervention in the field of exergaming. Before the project, the study and objectives of this research were presented to the school administration and then to the teachers. This pilot study took place in a small school with 1 class per level and 1 teacher per grade, with each of them (n=4) having no experience of exergaming and volunteering to take part in the research protocol. This school was selected for its pre-existing collaboration with the research team and middle-school students (8th grade), as well as for the availability of a space that could be used to install the LÜ mobile setup over a period of several weeks.

Subsequently, the parents of the children in the classes concerned were informed that their children would be part of a research protocol on 30 minutes of DPA during school time. A request for parental consent was sent via the school administration to each parent. In the event of parental refusal (only 3 parents refused), the children’s data were not analyzed. A habituation phase was then proposed, and the teachers were able to learn about the various potentialities of the LÜ tool, as well as the implementation of the 30 minutes of DPA by the project team. The intervention then began and lasted 3 weeks, and preintervention (T0) and postintervention (T1) assessments were conducted.

**Figure 1:**
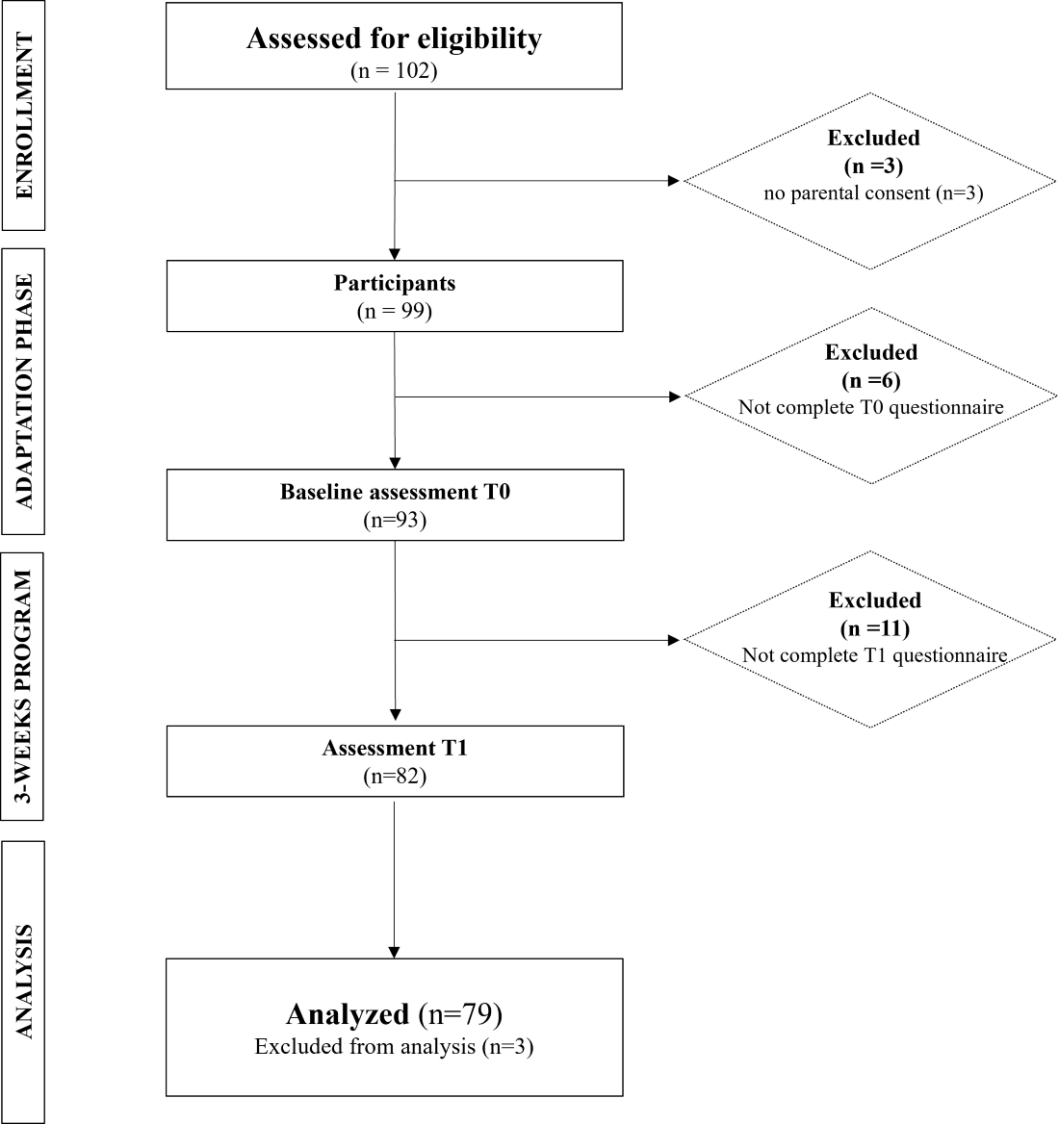
Study flow chart.

During the enrollment period, 102 children were eligible (Figure 1). However, owing to the absence of parental consent (n=3) or the absence of children at evaluation time 0 (n=6) or time 1 (n=11), the analyses were carried out on 79 children. This final sample was made up of 34 girls and 45 boys, with a mean age of 8.9 (SD 1.2) years.

### Class Measures

All the teachers expressed the wish to work on mathematics (geometry and arithmetic). A planning schedule was drawn up with the classes concerned so that the sessions could be scheduled during mathematics lessons.

### Teacher Measures

At the end of the program, teachers were asked the following questions: On a scale from 0 to 10, how would you rate (1) your students’ motivation for mathematics before the program? (2) your students’ concentration for mathematics before the program? (3) your students’ motivation for physical activity before the program? (4) your students’ motivation for mathematics today? (5) your students’ concentration for mathematics today? (6) your students’ motivation for physical activity today?

### Child Measures

At T0 and T1, students completed a questionnaire consisting mainly of analog visualization scales or checkboxes on different variables of interest (PL, motivation, self-efficacy, and concentration), which are described in the following sections. In addition, exercises adapted according to grade level were proposed in the target subject (mathematics) and a control subject (French).

#### Physical Literacy

PL was assessed using the Physical Literacy Assessment for Youth Self (PLAYself), designed for children aged 7 years or older, to explore children’s perceptions of their PL [35]. PLAYself demonstrated robust psychometric properties, with good fit statistics, internal reliability, and a lack of item bias and problematic local dependency [36]. For a better understanding of the different dimensions of PL in the PLAYself questionnaire, the forms are available in English [37] and French [38] versions. The adaptation of this form within the evaluation booklet of this pilot project is available in Multimedia Appendix 1.

PLAYself consists of 22 questions divided into the following four subsections: (1) *Fitness*, which involves children’s perceived fitness level with “disagree” and “agree” response categories for a single item; (2) *Environment*, which involves measures of 6 different environments in which children can do sports and activities (eg, “How good are you at doing sports and activities in the gym?”) on a 5-point Likert scale ranging from 1 (“never tried”) to 5 (“excellent”); (3) *Physical literacy self-description*, which involves 12 statements about doing sports and activities based on cognitive and affective factors (eg, “It doesn’t take me long to learn new skills, sports, or activities”), where the children are asked to rank how well they agree on a 4-point Likert scale ranging from 1 (“not true at all”) to 4 (“very true”); and (4) *Relative ranking of literacies*, which involves children’s ranking of the importance of literacies in school, at home with family, and with friends (eg, “Math and numbers are very important in school”) on a 4-point Likert scale ranging from 1 (“strongly disagree”) to 4 (“strongly agree”).

The first section is informative, while in the other 3 subsections, a separate score can be calculated and a total score can be obtained for PLAYself. The total PLAYself score is the average across the scores of each subsection, excluding the fitness question. A higher score (range 0-100) indicates a higher self-perceived PL.

#### Academic Achievement

To measure academic achievement, exercises in French and mathematics were retrieved from the national program by level following teacher school year progression. The test evaluated students’ academic knowledge and skills related to specific subject areas, including French and mathematics. The test was grade-specific; did not contain any bias regarding age, gender, or ethnicity; and was scored as a percentage of achievement in French on one side and mathematics on the other.

#### Motivation, Self-efficacy, and Concentration

Motivation and self-efficacy were assessed by 2 items each, one for mathematics and the other for French. For motivation, children were asked: “How much do you enjoy doing [mathematics/French] exercises?” For self-efficacy, children were asked: “How well do you think you did on the [mathematics/French] exercises?” Concentration was assessed by 3 items, one for mathematics, one for French, and a more general one targeting concentration in class. For this variable, children were asked: “How easy is it to concentrate in [class/mathematics/French]?” We used a simple question per variable to reduce the time needed to complete the entire protocol. The items were formulated as clearly as possible to be adapted to the children’s age and to ensure that they measure the core component of each variable. For all items, children were asked to respond using a 10-cm–long visual analog scale representing their feelings and marked by extreme labels at 0 cm (eg, very hard) and 10 cm (eg, very easy), which appeared as reliable response options in children’s questionnaires [39].

### Procedure

#### Habituation Phase

**Figure 2:**
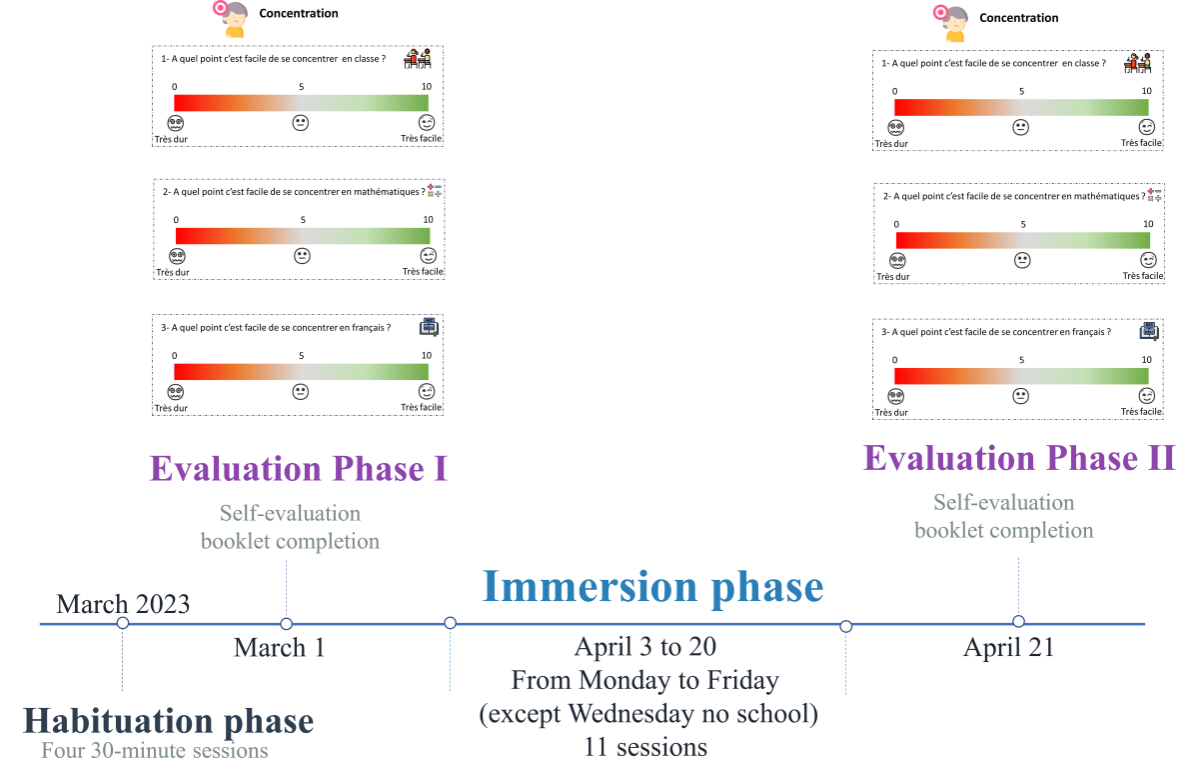
Timeline of the project.

On Thursdays in March 2023, students had 4 LÜ 30-minute habituation sessions, spaced 1 week apart, enabling them to familiarize themselves with the interactive gymnasium. Activities linked to the academic development of the LÜ catalog were proposed, targeting language (ie, Minewörd), mathematics (ie, Wäk, Newton, Constello, and SphYnX), science and technology (ie, Brüsh and Grüb), history (ie, Störia), and arts (ie, Pixël). With mathematics accounting for one-fifth of the school program in each grade and the LÜ catalog offering more mathematics-related applications (except PE, which was not at the center of the project), the 4 teachers wanted to work on mathematics during the 3-week DPA immersion phase (Figure 2).

#### Immersion Phase

After the habituation phase, students took part in 30-minute DPA sessions using the Play LÜ exergame platform and worked on a single subject selected by the teacher (Figure 2).

#### Exergame Setting

**Figure 3:**
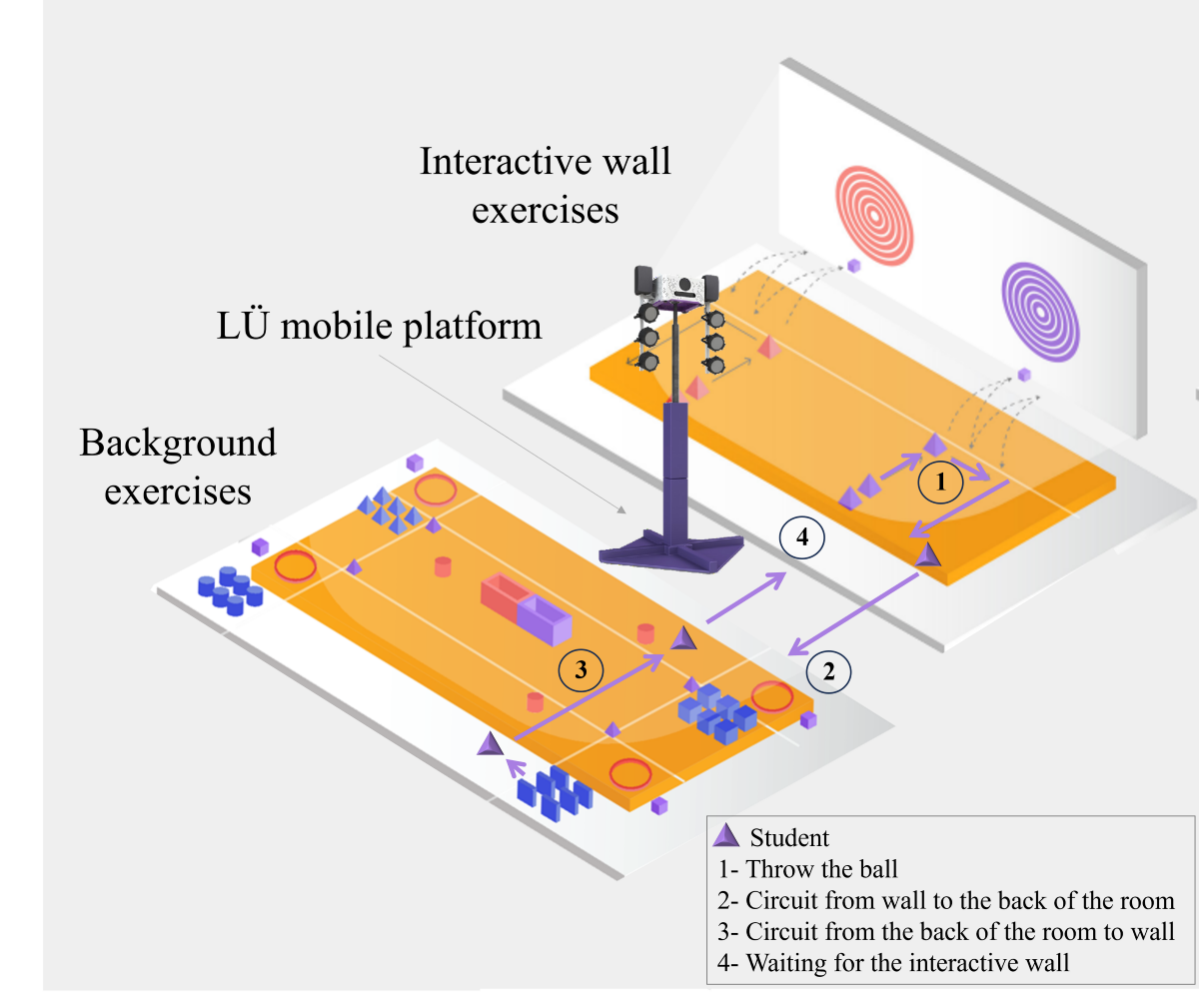
Practice environment implementation.

For this research protocol, the LÜ mobile equipment owned by the research team was made available to the school for this pilot project and was installed in a designated space for the duration of the project. The Play LÜ exergame platform (LÜ Interactive Playground) has the potential to overcome the limitations of a physical room. With Play LÜ, the participants are immersed in the games displayed on a giant projection wall (6×3 m). The principal mechanism of Play LÜ requires the players to throw balls against the wall (eg, to reach a target or to activate an object; Step 1 in Figure 3). In addition, this mobile platform offers an interactive game area for educational activities linked to specific learning themes (calculations, puzzles, etc). For this research protocol, a work area with a daily changing activity circuit was implemented behind the LÜ mobile setup (without the interactive wall). With class compositions ranging from 26 to 30 students, this “with” and “without” interactive wall configuration was essential to keep students active during the 30-minute session. Once the ball was sent to the interactive wall (Step 1), the student was required to go behind the LÜ mobile setup toward the back of the room (Step 2) to carry out various exercises to promote different motor skills (eg, jumping, throwing, and balancing) and perform other exercises on the way back (Step 3). At the end of the circuit behind the LÜ setup (Steps 2 and 3), the student waited for his or her turn in front of the interactive wall (Step 4).

#### Daily Session Exercises With Play LÜ

**Figure 4:**
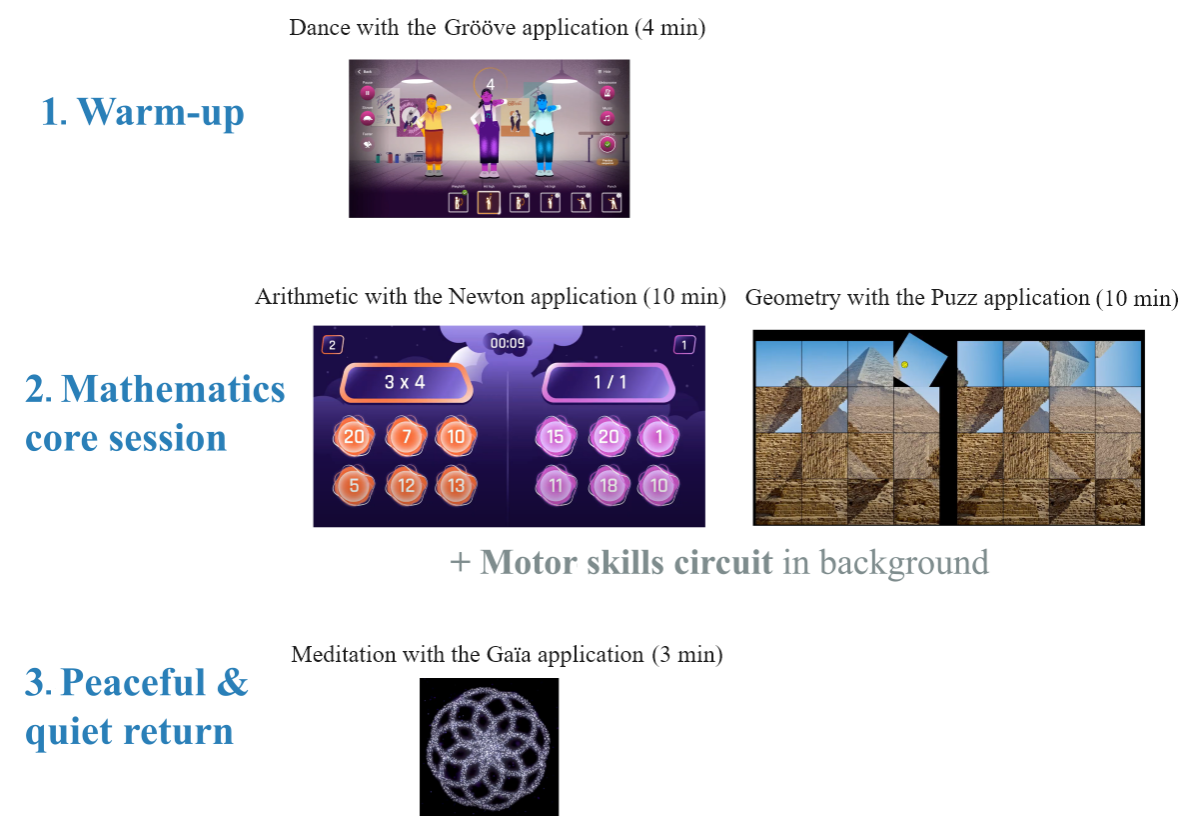
Daily physical activity core session details.

During the 3-week immersion phase, sessions were structured in the same way, with a warm-up using the Grööve application at the beginning (Figure 4), which can be assimilated with the active video game Just Dance (Nintendo) [40], and then a core session promoting mathematics learning adapted to each grade, involving a section in front of the interactive wall with the Newton application for arithmetic and Puzz application for geometry (ie, with picture geometric forms or rules), and a section without the interactive wall consisting of a motor skills circuit (eg, throwing, jumping, and balancing) that enabled the child to be as active as possible (Figure 4). During break times (mainly while waiting for their turn on the interactive wall), the children had access to posters presenting active health behaviors with their favorite heroes according to age (ie, The Minions, Miraculous, and a successful French singer or Youtuber, depending on student age). The session ended with a 3-minute meditation for a return to peace and quiet, using the Gaïa application (Figure 4).

#### Focus on LÜ Applications

The Play LÜ exergame platform allows the use of applications (with or without customization) that can be used to meet general or specific learning objectives. The applications used during the immersion phase and their pedagogical benefits are summarized in Table 1.

**Table 1:** Play LÜ applications.

Application	Duration (min)	Description	Learning objectives		
			Academic development	Physical and motor development	Sociocognitive development
Grööve	4	This is a perfect warm-up and allows the development of gross motor skills.	Movement Dance	Coordination Cardiovascular endurance	Cognitive flexibility Inhibition
Newton	10	Newton is a fun way to combine physical activity and mathematics with the customization of equations by grade.	Arithmetic	Manipulation (throw the ball and aim at the target)	Inhibition
Puzz	10	Throw the ball at a piece of the puzzle to rotate it and allow it to create an active knowledge competition.	Puzzle created with geometry-related images Spatial orientation	Manipulation (throw the ball and aim at the target)	Task engagement
Gaïa	3-5	This application helps students cool down after they have been active.	Health and healthy habits	Proprioception	Emotional regulation

The customization of pre-existing games in LÜ was carried out on the Newton and Puzz applications for mathematics learning. For the Newton application, the difficulty of the operations proposed depended on each grade (eg, addition and subtraction calculations for grades 2 and 3, and multiplication calculations for grades 4 and 5). For the Puzz application, the images to be assembled were linked to the geometry program (eg, polygons and nonpolygons for grades 2 and 3, and complex polygons for grades 4 and 5).

#### Evaluation Phase

Before (T0) and after (T1) the 3 weeks of DPA (Figure 2), students completed a self-evaluation booklet to assess their levels of PL, academic performance, and soft skills that could be impacted by the program (eg, motivation, concentration, and self-efficacy).

At the end of the 3-week immersion phase, the students retook the same questionnaire, with only minor changes to the exercises (eg, 12+18 replaced by 14+15), but with the same instructions and level of difficulty. Following this, a short interview was conducted with the teachers, asking them to assess the changes observed in their classrooms.

All DPA sessions were carried out by sports science students specializing in adapted PA and health, under the supervision of a qualified teacher of adapted PA and health.

### Statistical Analysis

Power analyses were conducted using G*Power (Heinrich-Heine-Universität Düsseldorf). For a pre-post comparison, with a medium effect size (0.50), an alpha error probability of .05, and a power of 0.95, we obtained a total sample size of 45. We then adjusted according to the number of participants available in the school, which allowed us to reach the sample size of 45.

As our data did not follow a normal distribution and given the characteristics of our sample, the intervention impact was tested with nonparametric within-group comparisons (T0 vs T1; Wilcoxon test, bilateral *P* values) for all participants and then by school-grade grouping. Effect sizes were expressed as the rank biserial correlation (*r*_rb_) and its 95% CI. We also provided the score differences between T1 and T0, and expressed them as a percentage of improvement. Data were analyzed using JASP software (version 0.17.2.1; JASP Team).

### Ethical Considerations

This pilot study involved an experiment in human and social sciences in the field of health. As mentioned in article R1121-1 section II subsection D of the French Public Health Code, this type of experimentation in human and social sciences does not require the authorization of the Committee for the Protection of Persons. Before the start of the study, a favorable opinion was obtained from the president of the University of Nîmes ethics committee. This individual verified that the study was conducted in accordance with institutional and national ethical standards, as well as the Declaration of Helsinki (2008). Moreover, this study was integrated into the school’s activities and projects, and the protocol was validated by the school administration.

### Consent to Participate

Concerning consent and information, the study was first presented to the school’s teaching staff and administration who gave their approval to take part. Next, an online information notice and online informed consent form with the names and university affiliations of the experimenters were provided to the parents of all children in the classes involved in the study before initiation. Finally, the information and informed consent of the children and their teachers were collected face-to-face. Recruitment was based on voluntary participation, with no compensation for participants. Participants were informed that they could withdraw their consent at any time, whether at the request of the child, parent, or teacher.

### Specific Measures Taken

To assure safety and security, all activities took place during class hours, under the supervision of the teacher, and the exercises were led by an associate professor specializing in public health and PA and two 3rd-year students in adapted PA from the University of Nimes. The expertise of the 3 animators enabled them to adapt the PA to the children’s abilities in order to prevent any risk of injury. Moreover, the number of animators made it possible to provide individual support when needed. Finally, to guarantee the security of the data, they were stored on a secure university computer, and the printed versions of the data were kept in a secure cupboard in a university office.

## Results

### Feasibility of DPA and Exergaming

With regard to our first objective, which was to study the feasibility of implementing 30 minutes of DPA through exergaming, our results showed that implementing exergaming during school time is entirely feasible. First, regarding the parents, all but 3 were in favor of their children taking part in the project. Second, regarding the teachers, all agreed to take part in the project. Third, all scheduled sessions (n=11) were carried out, with no sessions canceled. External constraints, such as educational visits or other activities, could have led to cancellations, but the teachers expressed a desire not to miss any sessions and agreed to exchange schedules with other classes when constraints arose, demonstrating their interest.

### Effects of the Exergaming Program

#### Effects on the Entire Cohort

With regard to our second objective for the whole cohort, the 3-week intervention of DPA led to increased scores in PL (in accordance with hypothesis 1) and motivation in mathematics, which was the subject covered in the intervention (in accordance with hypothesis 3). There was a general improvement regarding concentration in class, and we expected (hypothesis 5) this increase to be observed for mathematics as well (Table 2). In addition, contrary to our assumptions, we did not observe any changes in academic performance (hypothesis 2) or feelings of self-efficacy (hypothesis 4) in mathematics. Surprisingly, the intervention also favored French learning, which was not covered in the intervention, with academic performance, concentration, and self-efficacy in French being higher after the intervention.

**Table 2:** Descriptive analyses of variables of interest before (T0) vs after (T1) the 3 weeks of daily physical activity in the whole cohort (N=79).

Variable		Score before the program (T0), mean (SD)	Score after the program (T1), mean (SD)	Difference (T1-T0), value (% change)	*W*	*P* value	*r*_rb_^a^, value (95% CI)
Physical literacy		70.8 (13.6)	73.4 (13.3)	+2.6 (+3.6%)	933.0	.002^b^	−0.39 (−0.58 to −0.16)
**Academic achievement**							
	Mathematics	63.6 (32.5)	60.8 (31.0)	−2.8 (−4.4%)	1440.0	.35	0.12 (−0.13 to 0.37)
	French	59.3 (26.2)	65.0 (27.9)	+5.7 (+9.6%)	858.0	.05^b^	−0.26 (−0.50 to 0.00)
**Motivation**							
	Mathematics	7.1 (3.6)	7.8 (3.0)	+0.7 (+9.8%)	381.5	.005^b^	−0.44 (−0.66 to −0.16)
	French	5.3 (3.2)	5.6 (2.9)	+0.3 (+5.6%)	1222.0	.48	−0.09 (−0.34 to 0.16)
**Concentration**							
	Classroom (general)	5.5 (3.0)	6.0 (2.7)	+0.5 (+9.0%)	934.5	.07	−0.24 (−0.48 to 0.01)
	Mathematics	7.0 (3.2)	7.2 (2.8)	+0.2 (+2.8%)	580.0	.16	−0.21 (−0.48 to 0.08)
	French	5.6 (2.7)	6.4 (2.8)	+0.8 (+14.2%)	838.0	.01^b^	−0.34 (−0.55 to −0.09)
**Self-efficacy**							
	Mathematics	6.8 (2.8)	7.2 (3.1)	+0.4 (+5.8%)	887.5	.30	−0.14 (−0.40 to 0.13)
	French	6.1 (3.1)	6.7 (2.8)	+0.6 (+9.8%)	728.5	.02^b^	−0.32 (−0.54 to −0.05)

^a^Rank biserial correlation.

^b^Statistically significant.

#### Effects on Grade 5 Participants

Focusing specifically on each grade (see Multimedia Appendix 2 for full details), participants in grade 5 were those most affected by the intervention.

After the intervention, grade 5 participants showed an increase in PL (difference +5.6, percentage change +7.9%; *W*=9.0; *P*<.001; *r*_rb_=−0.91, 95% CI −0.96 to −0.78; hypothesis 1), mathematics motivation (+1.1, +20.0%; *W*=15.5; *P*=.007; *r*_rb_=−0.77, 95% CI −0.91 to −0.43; hypothesis 3), and mathematics concentration (+0.8, +12.7%; *W*=21.5; *P*=.01; *r*_rb_=−0.68, 95% CI −0.88 to −0.27; hypothesis 5) scores. Regarding French classes (even though they were not targeted by the intervention), grade 5 participants demonstrated an increase in motivation (+0.8, +17.7%; *W*=60.0; *P*=.05; *r*_rb_=−0.48, 95% CI −0.76 to −0.03) and self-efficacy (+1.5, +29.4%; *W*=45.0; *P*=.02; *r*_rb_=−0.57, 95% CI −0.81 to −0.14) scores after the intervention. They also tended to show an increase in academic achievement scores in French after the intervention (+7.3, +11.4%; *W*=53.0; *P*=.09; *r*_rb_=−0.44, 95% CI −0.75 to −0.03). Contrary to hypothesis 1, grade 5 participants showed a decrease in academic achievement scores in mathematics after the intervention (−10.7, −13.6%; *W*=192.0; *P*=.006; *r*_rb_=0.66, 95% CI 0.29 to 0.85).

#### Effects on Grade 2 to 4 Participants

The other grades also benefited from the intervention but to a lesser extent. After the intervention, grade 2 participants showed an increase in PL scores (difference +5.2, percentage change +7.1%; *W*=28.0; *P*=.03; *r*_rb_=−0.58, 95% CI −0.84 to −0.11; hypothesis 1) and a marginal increase in academic performance in mathematics (+15.1, +33.7%; *W*=18.5; *P*=.06; *r*_rb_=−0.59, 95% CI −0.86 to −0.06; hypothesis 2). Grade 3 participants showed an increase in mathematics concentration (+0.5, +7.5%; *W*=50.5; *P*=.04; *r*_rb_=−0.51, 95% CI −0.79 to −0.07; hypothesis 5) after the intervention. Regarding French classes, grade 2 participants showed a marginal increase in self-efficacy scores (+1.0, +14.4%; *W*=24.0; *P*=.07; *r*_rb_=−0.54, 95% CI −0.83 to −0.01), grade 3 participants showed a significant increase in academic performance (+16.8, +40.1%; *W*=68.0; *P*=.01; *r*_rb_=−0.58, 95% CI −0.80 to −0.21), and grade 4 participants showed a marginal increase in concentration (+1.1, +20.7%; *W*=25.0; *P*=.09; *r*_rb_=−0.52, 95% CI −0.82 to 0.01) after the intervention. Grade 4 participants showed a decrease in academic performance in French after the intervention (−15.0, −19.8%; *W*=103.5; *P*=.01; *r*_rb_=0.72, 95% CI 0.33 to 0.90).

#### Effects on Teachers’ Perceptions

Concerning the results obtained from teachers, 3 out of 4 teachers observed an improvement in their students’ motivation for mathematics after the program (grade 2, T0=5.5 vs T1=7.5; grade 3, T0=7.0 vs T1=7.5; grade 5, T0=8.0 vs T1=8.5), and 1 teacher observed no change (grade 4, T0=6.0 vs T1=6.0). In addition, 2 out of 4 teachers observed an improvement in their students’ concentration for mathematics after the program (grade 2, T0=6.0 vs T1=6.5; grade 4, T0=3.0 vs T1=5.0), and the other 2 teachers observed no change (grade 3, T0=6.0 vs T1=6.0; grade 5, T0=6.5 vs T1=6.5). Finally, regarding students’ motivation for PA, only 1 teacher observed an increase (grade 2, T0=8.0 vs T1=9.0). It should be noted that 2 of the teachers who observed no change (grades 4 and 5) had already identified the maximum motivation score before the start of the program. The last teacher rated the students’ motivation at 9.0 before and after the program.

## Discussion

### Principal Findings

Since September 2022, primary schools have been required to provide 30 minutes of DPA. In this pilot study, the implementation of an exergaming program as part of the 30 minutes of DPA policy was welcomed by the school’s administration, parents, and teachers, with an increase in perceived motivation for mathematics.

After the program, we observed that children showed increased scores in PL and motivation in mathematics following 11 learning-based exergaming sessions.

### Exergaming Implementation

Although this PA reform is recent in France, it has already been introduced in other countries several years ago. Indeed, the DPA school policy has been implemented since 2005 in Canada to promote active lifestyles for children in school settings [41], and in the province of Ontario, all elementary school children perform DPA during instructional time [42]. Yet, 10 years later (in 2015), it was revealed that only half of Ontario teachers were meeting this expectation [43], and this number dropped to 23% 5 years later in the report by Martyn et al [44]. The Canadian experience underscores the need to explore effective and sustainable methods for implementing the 30 minutes of DPA in schools. Consequently, this pilot study shows that exergaming can be used as a valuable tool in the deployment of DPA at schools.

### Efficiency and Usefulness of Exergaming

Our second objective was to find out whether an exergaming intervention could be effective and useful. We hypothesized that implementing a DPA program involving exergaming on a specific academic course could have an impact on different aspects of a child’s experience. First, in line with hypothesis 1, our results showed a significant increase in the PL of the entire cohort, with significant increases of over 7% for both grades 2 and 5. This result is all the more important as it has been highlighted that elevated PL leads to greater PA participation, resulting in positive physiological, social, and psychosocial adaptations, and thus improved physical, mental, and social health [45]. In other words, PL could play a role across the lifespan in promoting positive health. Therefore, exergaming seems to be an effective and useful instrument to promote PL. This observation is in line with the review by Sun [46], which highlighted that active video gaming could contribute to enhancing children’s PL, in particular on the motivational aspect of exergaming, making it possible to provide a variety of opportunities to develop or reinforce basic motor skills among children.

Second, concerning motivational aspects, in line with hypothesis 3, our results showed an overall positive effect on students’ motivation toward the discipline. Indeed, we found an increase in motivation for mathematics (target subject), with a significant increase of almost 10% for the total cohort, while motivation for French (control subject) was not impacted. It seems that allowing students to work on mathematics more entertainingly (ie, by throwing balls onto calculation operations) helps to increase their appeal in this course.

Third, contrary to hypotheses 2, 4, and 5, our results showed no increase in academic performance, motivation, and sense of efficacy in mathematics, but they showed an increase in these variables in French, even though this subject was not directly targeted in the sessions. Although the interpretation is limited without a control group that did not benefit from the intervention, it is conceivable that the participants benefited from additional motivational resources provided by the 30 minutes of DPA toward learning at school, in accordance with the results of Vazou et al [16] regarding motivation and self-efficacy. An argument in support of this explanation may be the marginal increase in general concentration in class for the total cohort, as has been observed in the review by Taras [47], which noted an immediate increase in concentration in students after PA. This overall concentration may have benefited all subjects, especially those frequently considered less difficult than mathematics (ie, French).

Finally, it is important to note that the positive effects of the intervention were found in all school grades, even if a greater benefit was observed in grade 5. As the ability to apply skills or knowledge learned during one activity to another activity is evidence of a transfer process, older children are likely to be more sensitive to it [48]. Indeed, in this study’s intervention, the children were learning with different tasks and objectives (in DPA exergaming and their normal lessons). The transfer of skills from one to the other was therefore not obvious (even if the “mathematics” cue was common to both) and remains a particularly demanding cognitive process for which the children need to be motivated. Decreases in academic performance in French and mathematics (grades 4 and 5) may be explained by constraints in the classrooms, as the teachers were rotated during the semester and the last data collection took place the day before the vacation (the participants were less involved overall in the academic exercises required). However, the marginal increase in mathematics performance in grade 2 and the increase in French performance (overall cohort and grade 3) demonstrate the importance of continuing to test this intervention.

### Practical Implications in the Educational Context

Learning-based exergames can be powerful allies in the implementation of the DPA policy at schools. For schools and educational teams, the first obstacle could be the associated cost. In France, the Ministry of Education has launched a call for projects entitled, “Pour un socle numérique dans les écoles élémentaires” (“for a digital base in elementary schools”) [49] to equip the schools of tomorrow. In this context, it is necessary to create links between the worlds of research and education. Researchers need to present teachers with the advantages (ie, academic performance, self-efficacy, motivation, PL, PA, and sedentary behavior) and constraints (ie, update, group management, and security) of this type of practice to make the teachers as efficient as possible in different teaching situations. Indeed, as part of the 30 minutes of DPA policy in elementary schools, one of the major difficulties is sustaining the actions and motivation of teachers, as presented in the Canadian study [43,44]. Once the equipment has been acquired and installed in a fixed position (ceiling-mounted model), one of the solutions for maintaining motivation among teaching teams would be to integrate PA professionals into the internship framework. This option enables teachers to not only benefit from the specific skills of the trainees but also position themselves as observers of the class, to be able to work on specific notions during PE teaching [18].

### Limitations

In the context of this pilot study, which focused mainly on implementation feasibility and learning, it would have been interesting to consider the children’s physical fitness (ie, muscular strength, agility, and cardiorespiratory fitness) and general state of health. Indeed, a French longitudinal study with a 3-year follow-up of children aged 7.7 years at the start of the study showed that the physical fitness of French youth decreased between childhood and early adolescence [50].

It would also have been interesting to compare the effects of this program with a control group. For example, it would have been worthwhile to compare the scores of the experimental group involving exergaming and targeted school exercises to 2 control groups: the first one with no PA and no school exercises, and the second one with no PA but with school exercises identical to the experimental group (eg, on a tablet computer). To verify the validity of the results, it would also have been necessary to vary the targeted school exercises (eg, mathematics and French; randomizing their inclusion in the intervention to ensure that the most difficult material is not the only one tested). Moreover, this study was carried out in a single school with a single class per level. It would be worthwhile to increase the size of the cohort by increasing the number of classes per level in different schools. Furthermore, our program had a limited duration (3 weeks), and a longer program (at least 1 trimester) with more DPA sessions would undoubtedly have increased the effects we observed and allowed additional benefits to be observed. In future studies, it would be interesting to compare the effects of a short program like ours with those of a longer program, and this could shed light on the duration of effects through time (in particular, following longer interventions).

### Perspectives

Future studies could explore the possible diffusion effects of enhanced DPA interventions with or without exergaming on various PA indicators (eg, physical fitness, increased mobility by accelerometry, sedentary time and breaks, and increased implication in PE curricula at school). It would also be useful to conduct a longitudinal study to measure the impact of exergaming on not only PA and fitness levels but also the evolution of overweight and obesity in children.

Furthermore, in future studies, it could be relevant to assess intervention effects on students’ academic performance, motivation, and self-efficacy in specific academic courses during interventions, or general attitudes and performances in different courses. Finally, specifying various student profiles concerning these measures (eg, depending on the initial levels of PL and PA, and depending on age or grade) could provide information on the subgroups of children benefiting the most from such exergaming interventions. In addition to student characteristics, it might be useful to consider teacher characteristics (eg, attitudes toward exergaming) to better understand the individual and environmental factors likely to moderate the effects of such interventions.

### Conclusions

As part of the 30-minute implementation of DPA, the use of learning-based exergaming showed very interesting results in increasing PL as well as student motivation toward mathematics. Furthermore, supporting pedagogical teams with qualified teachers in PA has been proven to be beneficial for both students and staff.

With this encouraging pilot study, it is necessary to continue investigations by increasing the number of students per grade and to carry out research over a longer school period with a control group to confirm these results regarding the use of learning-based exergaming with Play LÜ within the framework of DPA.

## Appendix

Multimedia Appendix 1 PlaySELF adaptation questionnaire.

Multimedia Appendix 2 Effects of the exergaming program according to grade.

Multimedia Appendix 3 CONSORT-EHEALTH checklist (V 1.6.1).

## Abbreviations

DPA: daily physical activity
PA: physical activity
PE: physical education
PL: physical literacy
