# Supplementary material for: Exploring the Use of a Learning-Based Exergame to Enhance Physical Literacy, Soft Skills, and Academic Learning in School-Age Children: Pilot Interventional Study
Source: JMIR Serious Games. 2024 Feb 23;12:e53072. doi: 10.2196/53072 (PMC10924257; doi:10.2196/53072)
Supplement: Multimedia Appendix 1 [file games_v12i1e53072_app1.pdf]

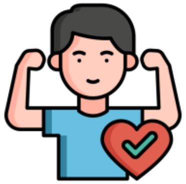

Ma condition physique est suffisamment bonne pour me permettre de faire toutes les activités que je souhaite faire.

Pas du tout vrai	Tout à fait vrai

1- Es-tu capable de faire du sport ou des activités …

	Jamais essayé	Plus ou moins bon	Bon	Très bon	Excellent
Dans le gymnase ? 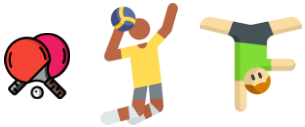					
A l’extérieur ? 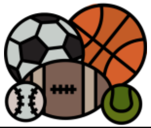					
Sur un terrain de jeux ? 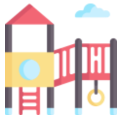					
Sur l’eau et dans l’eau ? 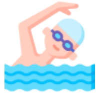					
Sur la glace ? 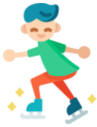					
Sur la neige ? 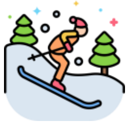					

Sport et activités physiques

**2- Que penses-tu du fait de pratiquer des sports ou des activités?**		Pas du tout vrai	Pas vrai	Vrai	Tout à fait vrai
1- J'apprends rapidement de nouveaux gestes, de nouveaux sports ou de nouvelles activités	1				
2- Je pense avoir les gestes nécessaires pour participer à n'importe quel sport ou activité si je le souhaite	2				
3- Je crois qu'il est important de bouger pour préserver ma santé	3				
4- Je crois que le fait de bouger me met de bonne humeur	4				
5- Je crois pouvoir participer à n'importe quel sport ou activité physique si je le souhaite	5				
6- Mon corps me permet de participer à n'importe quelle activité si je le souhaite	6				

Sport et activités physiques

2’- Que penses-tu du fait de pratiquer des sports ou des activités?

		Pas du tout vrai	Pas vrai	Vrai	Tout à fait vrai
7- J’angoisse à l’idée d’essayer un nouveau sport ou une nouvelle activité	7				
8- Je comprends les consignes données par les entraîneurs et les maîtresses	8				
9- J’ai confiance en mes capacités quand vient le temps de faire une activité	9				
10- Je suis toujours impatient(e) d’essayer une nouvelle activité ou un nouveau sport	10				
11- Je suis généralement le/la meilleur(e) de mon groupe quand il est question de faire une activité	11				
12- Je n’ai pas vraiment besoin de pratiquer. Je suis naturellement bon(ne)	12				

	Pas du tout d'accord	Pas d'accord	D'accord	Tout à fait d'accord
**La lecture et l'écriture sont très importants..**	☹	☹	☺	☺
A l'école				
A la maison				
Avec mes amis				

Les mathématiques sont très importantes …

A l'école				
A la maison				
Avec mes amis				

Les mouvements, les activités et les sports sont très importants…

A l'école				
A la maison				
Avec mes amis				

L'anglais est très important…

A l'école				
A la maison				
Avec mes amis				
