# Supplementary material for: Exploring the Use of a Learning-Based Exergame to Enhance Physical Literacy, Soft Skills, and Academic Learning in School-Age Children: Pilot Interventional Study
Source: JMIR Serious Games. 2024 Feb 23;12:e53072. doi: 10.2196/53072 (PMC10924257; doi:10.2196/53072)
Supplement: Multimedia Appendix 2 [file games_v12i1e53072_app2.docx]

**Multimedia Appendix 2.** Effects of the exergaming program according to grade.

Table S1: Descriptive analyses of variables of interest and before (T0) vs. after (T1) the 3 weeks of DPA comparisons for Grade 2.

|  | T0 | T1 | T1-T0 |  |  |  |
| --- | --- | --- | --- | --- | --- | --- |
|  | M (SD) | M (SD) | (% of improvement) | *W* | *P* | *R_rb_* [95% IC] |
|  |  |  |  |  |  |  |
| **Physical Literacy** |  |  |  |  |  |  |
|  | **72.6 (14.7)** | **77.8 (14.1)** | **+5.2(7.1%)** | **28.0** | **.03** | **-0.58 [-0.84,-0.11]** |
| **Academic achievement** |  |  |  |  |  |  |
| In mathematics | 44.8 (36.0) | 59.9 (32.6) | +15.1(33.7%) | 18.5 | .06 | -0.59 [-0.86,-0.06] |
| In French | 64.7 (21.7) | 71.0 (22.7) | +6.3(9.7%) | 12.5 | .25 | -0.44 [-0.83,0.25] |
| **Motivation** |  |  |  |  |  |  |
| In mathematics | 7.6 (3.6) | 8.8 (2.6) | +1.2(15.7%) | 13.0 | .28 | -0.42 [-0.82,0.27] |
| In French | 7.2 (3.2) | 6.6 (3.3) | -0.6 (-8.3%) | 58.5 | .38 | 0.28 [-0.31,0.72] |
| **Concentration** |  |  |  |  |  |  |
| In classroom (general) | 6.3 (3.4) | 7.3 (2.4) | +1.0(15.8%) | 32.5 | .38 | -0.28 [-0.72,0.31] |
| In mathematics | 8.6 (2.8) | 8.4 (2.3) | -0.2 (-2.3%) | 22.5 | .57 | 0.25 [-0.47,0.77] |
| In French | 6.4 (3.4) | 6.6 (2.7) | +0.2 (3.1%) | 44.5 | .97 | -0.02 [-0.56,0.53] |
| **Self-efficacy** |  |  |  |  |  |  |
| In mathematics | 7.7 (2.5) | 8.5 (2.7) | +0.8(10.3%) | 16.0 | .26 | -0.41 [-0.81,0.24] |
| In French | 6.9 (3.1) | 7.9 (2.5) | +1.0(14.4%) | 24.0 | .07 | -0.54 [-0.83,-0.01] |

Significant differences are specified in bold.

Table S2: Descriptive analyses of variables of interest and before (T0) vs. after (T1) the 3 weeks of DPA comparisons for Grade 3.

|  | T0 | T1 | T1-T0 |  | |  |  |
| --- | --- | --- | --- | --- | --- | --- | --- |
|  | M (SD) | M (SD) | (% of improvement) | *W* | | *P* | *R_rb_* [95% IC] |
|  |  |  |  |  | |  |  |
| **Physical Literacy** |  |  |  |  | |  |  |
|  | 70.7 (14.0) | 69.6 (13.3) | -1.1 (-1.5%) | | 182.5 | .86 | 0.04 [-0.38,0.44] |
| **Academic achievement** |  |  |  | |  |  |  |
| In mathematics | 54.2 (36.5) | 53.8 (30.9) | -0.4 (-0.7%) | | 147.0 | .79 | 0.06 [-0.38,0.48] |
| In French | **41.8 (27.9)** | **58.6 (34.6)** | **+16.8(40.1%)** | | **68.0** | **.01** | **-0.58 [-0.80,-0.21]** |
| **Motivation** |  |  |  | |  |  |  |
| In mathematics | 7.8 (3.0) | 8.2 (2.6) | +0.4(5.1%) | | 54.0 | .29 | -0.29 [-0.68,0.23] |
| In French | 5.0 (3.4) | 5.8 (2.8) | +0.8(16.0%) | | 136.5 | .49 | -0.16 [-0.54,0.27] |
| **Concentration** |  |  |  | |  |  |  |
| In classroom (general) | 4.6 (2.7) | 4.7 (2.6) | +0.1 (2.1%) | | 146.0 | .66 | -0.10 [-0.50,0.33] |
| In mathematics | **6.6 (3.1)** | **7.1 (2.5)** | **+0.5(7.5%)** | | **50.5** | **.04** | **-0.51 [-0.79,-0.07]** |
| In French | 5.1 (2.4) | 6.1 (2.9) | +1.0(19.6%) | | 94.0 | .11 | -0.37 [-0.69,0.06] |
| **Self-efficacy** |  |  |  | |  |  |  |
| In mathematics | 6.6 (3.1) | 6.9 (3.5) | +0.3 (4.5%) | | 103.0 | .67 | -0.10 [-0.53,0.36] |
| In French | 6.2 (3.4) | 5.8 (2.8) | -0.4(-6.4%) | | 97.0 | .95 | 0.02 [-0.45,0.48] |

Significant differences are specified in bold.

Table S3: Descriptive analyses of variables of interest and before (T0) vs. after (T1) the 3 weeks of DPA comparisons for Grade 4.

|  | T0 | T1 | T1-T0 |  |  |  |
| --- | --- | --- | --- | --- | --- | --- |
|  | M (SD) | M (SD) | (% of improvement) | *W* | *P* | *R_rb_* [95% IC] |
|  |  |  |  |  |  |  |
| **Physical Literacy** |  |  |  |  |  |  |
|  | 69.9 (11.8) | 71.7 (11.6) | +1.8(2.5%) | 58.0 | .62 | -0.14 [-0.60,0.38] |
| **Academic achievement** |  |  |  |  |  |  |
| In mathematics | 78.3 (21.6) | 64.0 (37.4) | -14.3(-18.2%) | 71.0 | .25 | 0.35 [-0.22,0.74] |
| In French | **75.7 (16.1)** | **60.7 (27.8)** | **-15.0(-19.8%)** | **103.5** | **.01** | **0.72 [0.33,0.90]** |
| **Motivation** |  |  |  |  |  |  |
| In mathematics | 7.3 (3.4) | 7.6 (3.2) | +0.3(4.1%) | 23.0 | .68 | -0.16 [-0.69,0.48] |
| In French | 4.9 (2.8) | 4.4 (3.0) | -0.5(-10.2%) | 62.0 | .57 | 0.18 [-0.39,0.65] |
| **Concentration** |  |  |  |  |  |  |
| In classroom (general) | 5.1 (3.2) | 5.7 (3.1) | +0.6(11.7%) | 32.0 | .61 | -0.17 [-0.67,0.42] |
| In mathematics | 7.3 (3.2) | 6.5 (3.4) | -0.8(-10.9%) | 46.0 | .06 | 0.67 [0.11,0.90] |
| In French | 5.3 (2.3) | 6.4 (2.9) | +1.1(20.7%) | 25.0 | .09 | -0.52 [-0.82,0.01] |
| **Self-efficacy** |  |  |  |  |  |  |
| In mathematics | 7.7 (2.8) | 7.1 (3.3) | -0.6(-7.7%) | 49.5 | .43 | 0.26 [-0.34,0.72] |
| In French | 6.6 (3.1) | 7.0 (3.4) | +0.4(6.0%) | 33.0 | .66 | -0.15 [-0.66,0.45] |

Significant differences are specified in bold.

Table S4: Descriptive analyses of variables of interest and before (T0) vs. after (T1) the 3 weeks of DPA comparisons for Grade 5.

|  | T0 | T1 | T1-T0 |  |  |  |
| --- | --- | --- | --- | --- | --- | --- |
|  | M (SD) | M (SD) | (% of improvement) | *W* | *P* | *R_rb_* [95% IC] |
|  |  |  |  |  |  |  |
| **Physical Literacy** |  |  |  |  |  |  |
|  | **70.3 (14.5)** | **75.9 (13.4)** | **+5.6(7.9%)** | **9.0** | **<.001** | **-0.91 [-0.96,-0.78]** |
| **Academic achievement** |  |  |  |  |  |  |
| In mathematics | **78.3 (18.5)** | **67.6 (24.1)** | **-10.7(-13.6%)** | **192.0** | **.006** | **0.66 [0.29,0.85]** |
| In French | 64.4 (22.4) | 71.8 (20.4) | +7.3 (11.4%) | 53.0 | .09 | -0.44 [-0.75,0.03] |
| **Motivation** |  |  |  |  |  |  |
| In mathematics | **5.5 (4.0)** | **6.6 (3.4)** | **+1.1(20.0%)** | **15.5** | **.007** | **-0.77 [-0.91,-0.43]** |
| In French | **4.5 (2.9)** | **5.3 (2.7)** | **+0.8(17.7%)** | **60.0** | **.05** | **-0.48 [-0.76,-0.03]** |
| **Concentration** |  |  |  |  |  |  |
| In classroom (general) | 6.2 (2.6) | 6.9 (1.8) | +0.7(11.2%) | 70.5 | .20 | -0.32 [-0.68,0.15] |
| In mathematics | **6.3 (3.3)** | **7.1 (2.8)** | **+0,8(12,7%)** | **21.5** | **.01** | **-0.68 [-0.88,-0.27]** |
| In French | 5.7 (2.9) | 6.5 (2.6) | +0.8(14.0%) | 61.0 | .10 | -0.41 [-0.73,0.05] |
| **Self-efficacy** |  |  |  |  |  |  |
| In mathematics | 5.6 (2.0) | 6.5 (2.9) | +0.9(16.0%) | 84.0 | .28 | -0.27 [-0.64,0.20] |
| In French | **5.1 (2.5)** | **6.6 (2.1)** | **+1.5(29.4%)** | **45.0** | **.02** | **-0.57 [-0.81,-0.14]** |
|  |  |  |  |  |  |  |

Significant differences are specified in bold.
